# Environment and Public Health: How the Environment Affects Children's Health and Quality of Life

**DOI:** 10.7759/cureus.78299

**Published:** 2025-01-31

**Authors:** Pinelopi Petropoulou

**Affiliations:** 1 Nursing, University of West Attica, Athens, GRC; 2 Dentistry - Periodontology, Dental Clinic "Synchroni Odontiatriki", Athens, GRC

**Keywords:** air pollution, children’s health, environment, public health, quality of life

## Abstract

The most well-known health effects of environmental degradation are related to air pollution, water quality, diet, poor sanitation, and hazardous chemicals. Air pollution is the biggest environmental health risk today. It affects the most vulnerable populations and is linked to heart diseases, strokes, lung diseases, lung cancer, and other organ diseases. Also, it affects neurodevelopment and cognitive abilities in children, can trigger childhood asthma and cancer, and can lead to chronic diseases such as heart attacks later in their lives. Indoor air pollution and the combustion of solid fuels lead to a large number of premature deaths from diseases caused by dangerous inhaled particles, especially in children. In addition, improper collection of waste that pollutes the soil, water, and atmosphere has been shown to be directly linked to public health, so the development and implementation of new waste management methods are imperative. With this work, we emphasize that children, in particular, have lifelong impacts on their health, educational prospects, and quality of life from air pollution and environmental degradation, and therefore monitoring, evaluation, and special planning for their health and well-being are required. The information gap needs to be immediately filled by raising awareness in society through educational programs in schools, the community, and the media, as well as by immediately implementing corresponding policies to achieve these goals.

## Introduction and background

By the term "environment," we mean the natural or artificial conditions in which someone or something exists and develops. Environmental health is the branch of public health concerned with all aspects of the natural and built environment affecting human health. The main risks facing environmental health today are the development of diseases caused by pollution and climate change, the way natural resources are managed, the production and management of excessive amounts of urban waste, noise pollution, water scarcity, and water pollution, and the radiation emitted by the industrialization of most societies. Epidemiological studies have documented the existence of short-term and long-term health effects from current levels of PM2.5, PM10, and ozone. Particulate matter (PM) or suspended particles (SP) are defined as any size of material in the air in solid or liquid form (from a few nm to a few tens of μm). Particles with d < 10 μm form the so-called aerosols. PM particles are usually measured in micrograms per cubic meter of air. PM2.5 covers all particles between 0 and 2.5 mm diameter and PM10 covers all particles between 0 and 10 mm. According to the World Health Organization, the permissible limit for PM10 particles that we can breathe is 50 micrograms/m3 while for PM2.5, it is 25 micrograms/m3 [1].

Air pollution can be just as dangerous as passive smoking; of course, active smoking is more dangerous for health. Living on busy roads carries about the same risk as passively smoking 10 cigarettes a day, and exercise should be avoided there during rush hour or when pollution levels are high. By reducing air pollution levels, it appears that the disease burden from stroke, heart disease, lung cancer as well as chronic and acute respiratory diseases can be reduced [1].

Free access to clean air is undoubtedly a fundamental human right. A person breathes an average of 10 million times a year. Children naturally breathe faster than adults, and when they live in a polluted environment, they absorb more pollutants. Household air pollution is a significant form of indoor air pollution mostly relating to cooking and heating methods used in developing countries. Since much of the cooking is carried out with biomass fuel, in the form of wood, charcoal, dung, and crop residue, in indoor environments that lack proper ventilation, millions of people, primarily women and children, face serious health risks. Household air pollution has been shown to increase the risk for a wide range of adverse cardiorespiratory, pediatric, and maternal outcomes, particularly in poorer low- and middle-income countries [1]. The right to health provides a valuable opportunity to engage different parties to advocate for climate action. The public debate on the climate crisis has so far failed to recognize the interconnected nature of these issues, and this study also aims for this purpose. Additionally, human rights literacy in the context of climate change needs to be strengthened within the public health community [2].

Air pollution affects the health of 17 million babies in the world. In addition, today's trends of removing children from nature create problems for them and their society [1]. It has also been shown that prenatal and early exposure to air pollution can lead to serious consequences, including the development of asthma in children in urban areas when exposed to nitrogen dioxide (NO2) in the long term. There are potential associations with many diseases and complications during the lifetime and the quality of life levels, especially in developing countries. Some examples are diabetes, low birth weight, premature births, cognitive decline and dementia, Parkinson's disease, the impairment of cognitive development in children, and poor mental health outcomes across the lifespan [3].

## Review

Aim - methodology

**Figure 1:**
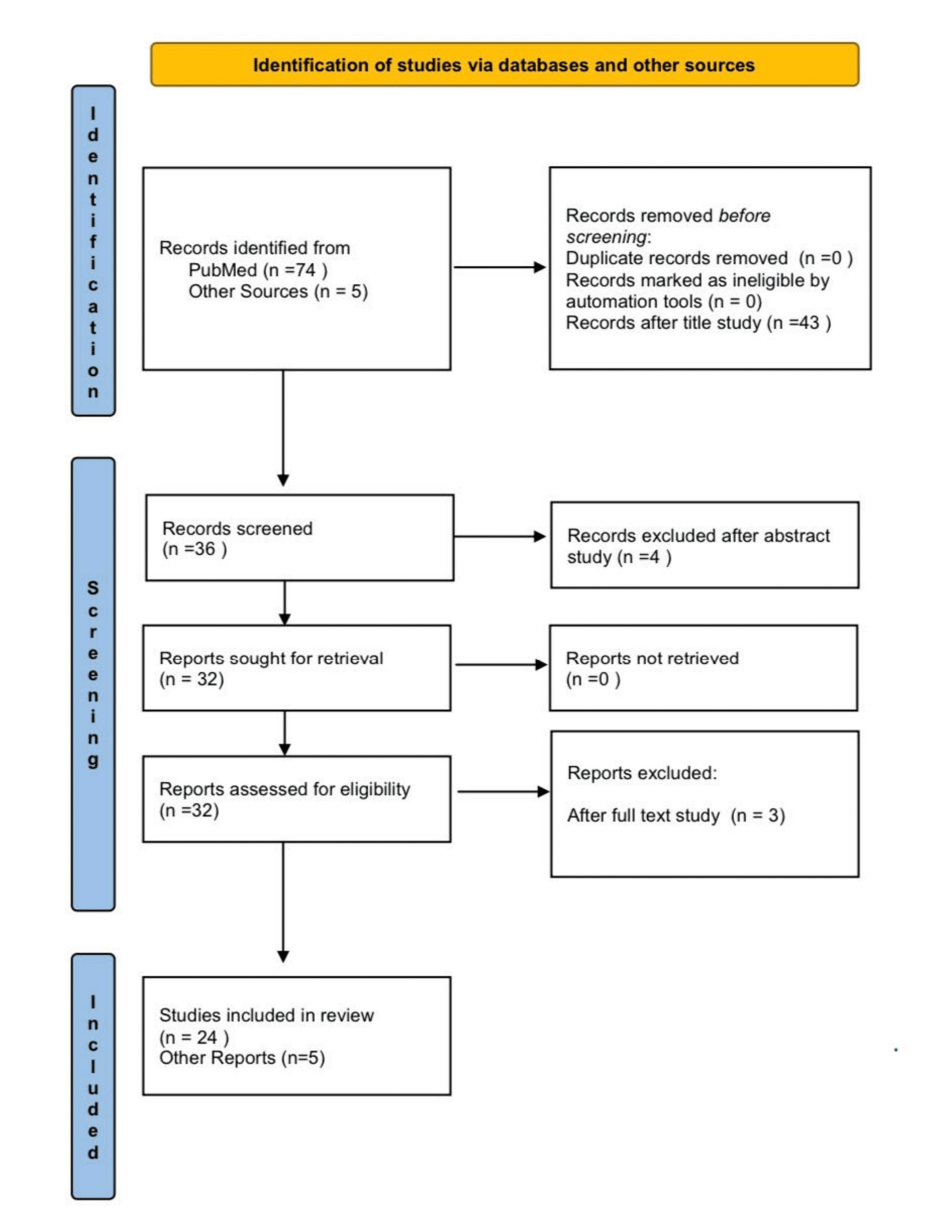
Flowchart showing the process of searching and locating research articles.

A narrative review was conducted with the purpose of highlighting the most frequent and serious lifelong impacts on the overall health and quality of life of children from environmental degradation to contribute to public awareness and the establishment of public and environmental health policies to prevent and address these problems. A search was conducted in the PubMed database, using Boolean operators with keywords, including ''air pollution'' AND ''child health'', with the full text of the study freely available through open access, for the period 2019-2024. A total of 74 articles were identified, of which 43 were rejected after studying their titles, and four articles were rejected after studying their abstracts. A total of 27 studies were studied and three were rejected after studying the full text. Finally, 24 articles were included in the present study according to the selection criteria (Figure 1). An in-depth analysis of the studies was carried out to obtain a list of the most relevant data on the subject.

The inclusion criteria for this descriptive study were as follows: (1) studies with full text available for study through open access; (2) studies conducted in the last five years (2019-2024); (3) studies written in English; (4) studies that refer to specific environmental health problems of the modern era; (5) studies that examine the most frequent and serious impacts on the overall health of children from environmental degradation and consequently on their quality of life; (6) studies that refer to proposals for prevention and treatment with public and environmental health measures.

Study limitations

The search was conducted only in the PubMed database, including studies written in English, with their full text freely available through open access, for the period from 2019 to 2024, and suitable according to the research inclusion criteria (Figure 1).

The following subsections describe the most frequent and most serious effects on children's health, based on research, from environmental degradation and especially from air pollution and hazardous chemicals.

Atmospheric pollution

Air pollution is the biggest environmental health risk in Europe today and is linked to heart disease, strokes, lung disease, and cancer of the lungs and other organs. Particularly affected are the vulnerable populations of women, pregnant women, children, the disabled, and the elderly, who also make up the majority of the world's poor. The foci of pollution are mainly factories, power stations, vehicles powered by fossil fuels, dust and waste burning, and air pollution often caused by coal while there seems to exist an association between benzene and childhood leukemia risk [1].

Acute leukemia is the most common childhood cancer and environmental factors seem to play a key role in its etiology. In addition to ionizing and non-ionizing radiation, infections, pesticides, parental smoking, diet, and occupation, there is also strong evidence for outdoor air pollution, particularly exposure to pollutants from motor traffic. Benzene exposure was associated positively and linearly with the risk of childhood leukemia, especially for acute myeloid leukemia, with no minimum exposure threshold, among children under six years of age while exposure to nitrogen dioxide (NO2) showed little association with the risk of leukemia except at the highest levels. Disease subtype, exposure time, and child age appear to modify these associations [4].

Traffic-related air pollution (TRAP) exposure might lead to lung function impairment and constant respiratory symptoms. Possible underlying mechanisms include lung epithelial injury, inflammation, oxidative stress, and energy metabolism disorders. Also, increased exposure to ambient air pollutants is associated with a higher risk of influenza-associated illness. Thus policies to reduce air pollution levels may also help reduce morbidity due to influenza infection [5].

In today's big cities, the air we breathe contains a variety of toxic substances and pollutants that come mainly from the combustion of hydrocarbons used for the movement of cars and industry. Road transport is the main source of NO2 emission, one of the main pollutants that harm our health. This pollutant is also a precursor compound of ozone and suspended particles (PM) that can form in the air. Studies showed that exposure to PM2.5, PM10, sulfur dioxide (SO2), and NO2 was associated with a significantly increased risk of gestational diabetes mellitus (GDM), especially in preconception and first trimester, while at the same time, there is a high correlation between NO2 and chronic obstructive pulmonary disease (COPD) mortality [6].

Both long-term maternal exposure in the second or third trimester and short-term maternal exposure to air pollution may increase the risk of premature rupture of membranes (PROM). Also, it seems that there is a significant statistical correlation between exposure to PM10, PM2.5, and O3 and the risk of the occurrence of orofacial clefts (OFCs) in the second month of pregnancy. Preterm birth, low birth weight, and autism have also been associated with maternal preconception exposure to PM2.5, PM10, O3, and SO2. Additionally, the potential effects of paternal preconception exposure need to be studied [7].

We know that toxic pollutants through the respiratory function can reach all the organs of the human body and cause disorders or impairment of their function, as a result of which they become vulnerable and burdened by other factors as well.

The World Health Organization (WHO) concluded that air pollution affects neurodevelopmental and cognitive abilities in children. It can also trigger asthma and childhood cancer [8]. Moreover, children exposed to elevated levels of air pollution could be more likely to develop chronic conditions such as cardiovascular disease later in life [9].

The International Commission on Environment in its research on children concluded that air pollution is directly linked to autism spectrum disorder (ASD). Research has shown that children exposed to high levels of air pollution are 78% more likely to develop ASD compared to a child living in low levels of air pollution [10].

Poor air quality also has significant economic impacts on health policies and leads to increasing medical care costs, reducing worker productivity, and damaging soil, crops, forests, lakes, and rivers [11].

The most common health effects related to air pollution from suspended particles (PM 2.5 µm) are pulmonary infection and an increase in COPD, hospital admissions, cardiovascular diseases, and mortality. Health effects from ozone (O3) are the impairment of respiratory function, the inhibition of lung development, the increase in morbidity and mortality from NO2, the increase in the incidence of asthma, allergic pulmonary reactions, and hospital admissions [12,13].

Reasons for the higher sensitivity of children to air pollution

The higher sensitivity of children to air pollution is due to some differences from adults, including physical differences, since children have less developed thermoregulatory systems, for example, a lower sweating rate and a higher body surface/mass ratio, which means a higher rate of heat exchange with the environment [14]; metabolic differences, since they have a higher metabolic rate than adults and therefore greater sensitivity to heat waves or frost [15]; cardiovascular differences, since they have a lower cardiac output than adults, a smaller total blood volume, and therefore less adaptability to large increases in temperature and pollution; behavioral differences, since they spend more time outdoors often with intense activities so their exposure to heat and pollutants is increased [9].

Naturally, they show a lack of self-care and are highly dependent on others and their environment. They have a longer life expectancy than adults, thus a longer exposure time to environmental conditions but also a longer time for the effects of this exposure to appear.

We also know that vigorous exercise and play increase the ventilation rate up to 17-fold in children, making vigorous play and exercise in children particularly dangerous in highly polluted areas [16,17].

A study of more than 3,500 children with no history of asthma in areas with high ozone concentrations showed that children who played three or more outdoor sports were up to 30% more likely to develop asthma than those who played no sports.

About 300 million children, or nearly one in seven children worldwide, live in areas where air pollution levels are, according to the United Nations Children's Fund (UNICEF), at least six times higher than the permissible limits set by the World Health Organization. UNICEF announced that air pollution is a "significant factor contributing to the death of approximately 600,000 children under the age of five each year" by causing diseases such as severe pneumonia. In addition, increased pollutants have been shown to make it difficult for children's lungs to develop, resulting in limiting the blood going to their brains and eventually causing permanent damage to their brain development [18].

Key findings of the World Health Organization

Air pollution affects the development of children's intellectual and motor skills (cognitive impairments) and damages the lung function of children, even if they are not exposed to high but lower levels of exposure. A total of 93% of children worldwide under the age of 15 years are exposed to particulate matter (PM2.5) levels above the WHO air quality guidelines.

More than 40% of people worldwide - including 1 billion children under the age of 15 - are exposed to elevated levels of indoor air pollution. The main causes are cooking with polluting technologies, the use of bad fuels, and old ‘’sick buildings.’’ About 600,000 deaths among children under 15 were attributed to the effects of both household and outdoor air pollution in 2016. A total of 160 million children were found in areas of high or extreme drought and over 500 million children in areas with very high frequency of flooding.

Malnutrition caused by climate change is predicted to lead to an increase of up to 130,000 deaths among children under five years of age in 2030 while harming children's developmental potential and health in the long term of their immune system.

Vigorous exercise increases the ventilation rate up to 17 times in children, which makes children's vigorous play and exercise particularly unsafe in highly polluted areas [11].

Indoor pollution - the sick building syndrome

According to the WHO, one in three new or renovated buildings has "indoor pollution" problems. The term "sick building" describes buildings intended to house services or residences, which present problems of indoor pollution. The air quality of these indoor spaces is considered poor. The main characteristic of "sick buildings" is poor indoor air quality. The main reason for the appearance of a sick building is insufficient or improper ventilation. We already know that each person usually spends 33% of their time indoors and it can be extended up to 66%. More studies show that particular attention needs to be paid to compounds that may promote or even enhance the development of adverse health problems. It is worth noting that the WHO includes formaldehyde (group 1 - human carcinogen) and monoxide of carbon (CO) as relevant indoor air pollutants, while monitoring particulate matter (PM), CO, and ozone (O3) is recommended for the presence of outdoor air inside. Consequently, PM10 and PM2.5 (particles with an aerodynamic diameter of 10 and 2.5 µm, respectively), total volatile organic compounds (TVOC), formaldehyde, CO, and O3 are among the compounds included in the list of pollutants to be monitored in indoor air. Indoor temperature (T) as well as relative humidity (RH) may also contribute to indoor pollutant accumulation. Most of the available studies have focused on children's exposure to indoor air pollutants in homes, nurseries, kindergartens, and primary schools [16,19].

The total time inside the indoor environment depends on the age of the people, their state of health, their type of work, the climate of the place they live in, as well as their habits. Newborns, babies, preschool children, and the elderly spend almost all of their time indoors and they are the ones most affected [20].

Indoor air can be contaminated with a mixture of air pollutants, hazardous chemicals, radon, and mold, leading to significant health effects while it is estimated that 2 million disability-adjusted life years are lost annually in the European Union (EU) due to poor indoor air quality.

Especially when polluting fuels or other technologies for cooking, lighting, and heating are regularly used in homes, such as fireplaces, cigarette smoke, kerosene heaters, gas stoves, petrol equipment, candles, and incense, children are much more sensitive to household air pollution. Mechanical or natural ventilation of the building must ensure suitable conditions in terms of temperature and humidity and maintain indoor pollutant concentrations at acceptable levels [21]. The symptoms of the sick building syndrome are the following: shortness of breath, dry cough, sore throat, runny nose, tearing, headaches, dizziness, nausea, mental fatigue and confusion, physical fatigue, lethargy, and digestive disorders. Efforts to improve indoor air quality require a comprehensive approach, including building design and management, structural products, and education to promote positive behavior [22].

We know that the use of solid fuels represents globally the largest source of indoor air pollution. The aromatic carbon rings/volatile organic compounds (VOCs) arising from it can cause acute and chronic health effects in building occupants, such as cancer, paralysis, lung failure, and more. Bacterial spores, fungal spores, mold spores, pollen, and viruses are types of biological contaminants and can cause allergic reactions or an illness described as the sick building syndrome. In addition, outdoor pollution, such as vehicle exhaust, can enter buildings, deteriorate indoor air quality, and increase indoor carbon monoxide and carbon dioxide concentrations. Moreover, indoor temperature below 18°C (64°F) has been shown to be associated with increased respiratory and cardiovascular disease, increased blood levels, and increased hospitalization [19].

To understand the magnitude of the problem, here is the typical data from the WHO and the United States Environmental Protection Agency (EPA) on the effects of indoor air pollution: about 3 billion people cook and heat their homes using fossil fuels in fireplaces and in stoves that leak combustion pollutants, and about 2.7 billion burn biomass (wood, animal manure, crop waste), and an additional 0.4 billion use coal.

Almost 2 million people annually die prematurely from diseases caused by indoor air pollution from burning solid fuels. Of these deaths, 44% are due to pneumonia, 54% due to COPD, and 2% due to lung cancer.

About 50% of childhood deaths from pneumonia are caused by inhaled particulate matter from indoor air pollution, while more than 1 million people die each year from COPD that they develop from exposure to polluted indoor air [11].

Methods of reducing indoor pollution

Indoor air pollution can be reduced by a number of measures, such as removing pollution sources from closed spaces or limiting their emissions; improving ventilation with the outside air when it is cleaner than the inside; cleaning indoor air by placing certain indoor plants, such as ivy, spiderwort, and aloe, which have the ability to trap formaldehyde, carbon monoxide, benzene, and other toxic gasses; avoiding storing solvents and other volatile substances indoors; contributing to reducing pollution by avoiding the use of synthetic carpets in indoor spaces that release over 100 types of VOCs and are hotbeds for the accumulation of microbes, dust, traces of lead, and pesticides, as well as avoiding the use of aerosols, sprays and air fresheners. Also, the ecological building and the bioclimatic design of buildings with the use of construction materials with low formaldehyde content, as in recent years, is supported and promoted by research by ecologists, architects, and engineers.

Bioclimatic architecture is a modern trend in building construction. Bioclimatic architecture is an innovative architectural approach that focuses on the construction of buildings that harmonize with the natural environment and the local climate, utilizing environmentally friendly practices. In the international literature, the term "smart building" prevails, which refers to the design of buildings and spaces with the aim of utilizing solar energy and other renewable energy sources as well as natural climatic phenomena.

The promotion of bioclimatic architecture aims to protect the environment and natural resources. The weaning from oil is achieved by trying to utilize alternative energy sources, mainly renewable sources, namely, the sun, wind, and water. Due to reduced consumption of oil and electricity more than 50% of the money is saved [19,21].

Waste management and disposal

There is global concern over the increase in solid waste generation due to intense urbanization and industrialization in recent years. Effective solid waste management is oriented toward both developed and developing countries due to its adverse effects and impacts on human health and the environment, respectively.

The improper collection of waste pollutes the soil, water, and the atmosphere. Very often the final disposal of this waste is done in landfills or in incinerators, which in turn pollute the atmosphere. Research on landfills has shown that conditions are often not favorable for biodegradation.

Leachate leakage from landfills contaminates soil and groundwater and therefore can have a serious impact on human health, especially when leachates are released uncontrollably. The presence of several pollutants, including suspended particulate matter (organic and inorganic), toxic metals (TMs), and heavy metals (HMs) in landfill leachates is of concern and may pose a serious threat to public health as well as ecotoxicological effects on terrestrial and aquatic ecosystems. HMs can accumulate in the food chain and then enter the human body through skin absorption, contact, direct ingestion, and inhalation, so children are at more risk than adults.

The carcinogenic process is initiated as a result of DNA damage in cells, caused by reactive oxygen species (ROS) because they can play an important role in metal-induced cellular responses. Attention and actions are needed to minimize and prevent the contamination of the soil, water, and air, from toxic and heavy metals, by slowing the movement of leachates through the appropriate design of new disposal sites with suitable foundations.

Government agencies should consider new technologies with better potential to remove these harmful metals from landfills to prevent their release into soil and water.

Waste incineration plants emit a number of gaseous derivatives such as fly ash and gaseous pollutants into the atmosphere, with significant impacts on air quality. Human exposure to dioxins from waste incineration has been shown to be associated with the risk of carcinogenesis; in addition, heavy metals at toxic waste sites as well as polycyclic aromatic hydrocarbons (PAH) formed during incomplete combustion of organic materials lead to human carcinogenesis such as in the lung, skin, and bladder, and leukemias in children.

Moreover, exposure to microplastics can also occur through inhaled air, which can affect important biological processes in the human body, and cause disruption of the endocrine and immune systems. Microplastics may have a negative impact on motility, reproduction, and development and can cause carcinogenesis.

As we mentioned above, the research shows that these results are more devastating for the health of vulnerable populations, especially children.

The implementation of new solid waste management methods is imperative. The answer seems to lie in tracking the entire life cycle of products "from birth to death." Finding ways to use fewer raw materials and more recyclable and reusable materials also means less waste. This will especially help children who are directly affected, as they spend a large part of their lives on the ground and outdoor atmosphere, where most of the pollutants from waste accumulate [19,23].

The waste management policy in each municipality must be based on the principle of participation and there must be continuous and open communication with all citizens to have a continuous and two-way dialogue, such as well-prepared public debates and actions at the neighborhood level. Moral and material support is needed for non-profit organizations present in the city to deal with special issues and problems such as cleanliness.

Dissemination of information for recycling with periodic publications and announcements from municipalities and regions and environmental education and health promotion in the country's schools is an imperative and immediate need for public health, contributing to the correct waste management behavior from new generations [24].

How children's mental health is affected by the environment

Children who grew up in an environment with a lot of greenery have a significantly lower risk of various mental disorders later in life, according to new scientific research, which highlights the importance of green spaces for people's mental health as well. The study shows that children who grew up surrounded by greenery had on average a 55% lower risk of developing a mental disorder later in life, even when burdened by other risk factors such as socioeconomic status or family history of mental problems [25].

The risk of developing a mental disorder decreases the more one is surrounded by greenery from birth to the age of 10 years. Therefore, the presence of greenery during childhood is very important and there is now growing evidence that the natural environment plays a greater role in mental health than previously thought. Physical exercise in the natural green environment has been shown to contribute to children's well-being and cognitive functions, as well as reducing obesity [25].

One of the most vulnerable groups to climate change is children due to their immature thermoregulatory functions, biological sensitivity, and limited adaptive responses to their environment. The fact that they will face the climate dilemma for many years combined with their existential concerns about the future in the face of alarm and social tension due to pandemics, such as Ebola or the coronavirus, can have unpleasant effects on the sensitive child psychology. Epidemiology and psychiatry in an interdisciplinary collaboration have a certain tradition of studies on psychosocial risk factors. Environmental risk factors such as noise, temperature, air quality, and toxic substances have been shown to contribute to mental morbidity and mortality [26].

Studies have found that more green spaces in an area create greater social cohesion, increase the physical activity levels of residents, and improve the mental development of children. During childhood, an active lifestyle is particularly important as it helps regulate children's metabolism and their physical and psychological health. Physical activities promote a person's functional status and mental health, thus improving their quality of life [27].

They also help shape the normal development of the musculoskeletal and immune systems, while at the same time improving their self-esteem, initiative, and academic performance. Τhe greater the children's exposure to green spaces both at home and at school, the better their cognitive performance. It promotes their socialization through play, the acquisition and cultivation of environmental awareness, and awareness of nature [28,29].

Table 1 summarizes the results of the studies included in our study, mentions the date and the country in which they were conducted, the type of each study, and the conclusions about health and quality of life in which they ended up.

**Table 1:** Characteristics of the included studies. NO2: nitrogen dioxide; O3: ozone; COPD: chronic obstructive pulmonary disease; ADHD: attention deficit hyperactivity disorder; GDM: gestational diabetes mellitus; PTSD: post-traumatic stress disorder; Cr: chromium; Mn: manganese; Cu: copper; As: arsenic; Cd: cadmium; Ba: barium; Hg: mercury; Pb: lead.

Authors/year/references	Type of study	Country		Methodology	Health results
Patterson (2021) [2]	Review article	Switzerland		This paper addresses the lacunae within the context of the right to health as enshrined in United Nations human rights treaties and related international law.	States’ obligations to address the climate crisis and concomitant health crisis from a right-to-health perspective.
Filippini et al. (2019) [4]	Systematic review	Italy		Case-control and cohort studies (n = 66) have investigated the risk of childhood leukemia in relation to exposure to motorized traffic and related contaminants. A dose-response meta-analysis was also performed to understand the shape of the curve relating to air pollution and disease risk (n = 29).	This study investigated the extent to which outdoor air pollution, especially as resulting from traffic-related contaminants, affects the risk of childhood leukemia.
Huangfu et al. (2020) [6]	Systematic review and meta-analysis	UK		46 cohort studies assessed long-term concentrations of NO2 and O3 and mortality. A meta-analysis of 24 studies found an increased risk of death associated with NO2.	Summary of the available evidence on the effect of long-term exposure to ozone (O3) and nitrogen dioxide (NO2) on mortality.
Chen et al. (2020) [7]	Systematic review and meta-analysis		The Netherlands	104 cohort studies from all over the world showed associations between long-term exposure to PM2.5 and PM10 in relation to all-cause and cause-specific mortality.	There is clear evidence that both PM2.5 and PM10 were associated with increased mortality from all causes, including cardiovascular disease, respiratory disease, and lung cancer.
Sordillo et al. (2019) [8]	Clinical trial		USA	996 mother-child pairs during the first and second trimesters of pregnancy, and both mothers and children at delivery and periodic postnatal research visits, using blood samples (p < 0.05).	The research identifies potential protective prenatal nutrients, as well as adverse prenatal pro-oxidant exposures that may alter the risk of asthma and allergic disease into adolescence.
van Meel et al. (2022) [9]	A meta-analysis of 150,000 European children		Europe	Data from 150,090 children primarily from the EU Child Cohort Network to examine the associations of upper and lower respiratory tract infections from age six months to five years.	Early-life upper respiratory tract infections are associated with an increased risk of school-age asthma. Early-life lower respiratory tract infections are associated with lower lung function at school age.
Krieger (2020) [11]	Review article		USA	Practical action steps for civic engagement and democratic governance to promote equitable and integrated policies to address the climate crisis and build health equity.	The public health and medical communities have provided critical evidence about the myriad adverse and highly inequitable health impacts of climate change.
Park et al. (2021) [12]	Systematic review and meta-analysis		South Korea	Of the 436 studies identified, six, three, and five had data on PM2.5, PM10, and NO2, respectively.	Long-term exposure to PM2.5 and NO2 is associated with an increased incidence of COPD.
Kim et al. (2018) [13]	Meta-analysis		Korea	A total of 30 cohort studies were included in the final analysis. Examination of the relationship between main air pollutants and all cancer mortality.	Data showing an association between air pollution and all-cancer mortality have important implications for public health.
Fuertes et al. (2020) [14]	A meta-analysis of European birth cohorts		Europe	Current eczema, rhinoconjunctivitis, and asthma were assessed in children aged four (N = 6527) and eight years (N = 2489).	Associations of long-term air pollution levels at home with pediatric eczema, rhinoconjunctivitis, and asthma prevalence in five birth cohorts.
Wang et al. (2020) [15]	A systematic review and meta-analysis		China	39 studies are selected with environmental factors of interest, including traffic flow, traffic pollution, traffic noise, and traffic safety.	Long-term traffic pollution is positively associated with children's BMI growth and traffic flow, pollution, and noise could affect weight-related behaviors.
Saridi et al. (2021) [16]	Clinical trial		Greece	This study investigates the impact of indoor air pollution on children's health, questioning 126 parents of children of preschool age (p < 0.05).	Improving indoor air quality is necessary, given the negative effects of pollution on human health, especially on the more vulnerable groups, such as children.
Urrutia-Pereira et al. (2022) [17]	Review article		Brazil	A non-systematic review of English, Spanish, and Portuguese articles published in the last five years in databases such as PubMed, Embase, and SciELO.	The study documented that prenatal and postnatal exposure to ambient air pollutants will accelerate or worsen the morbidity and mortality of many health conditions in children.
Hao et al. (2023) [18]	Clinical trial		USA	Evidence on effects of air pollution on adverse birth outcomes and pregnancy complications (at least 27 weeks of pregnancy) (p < 0.05).	Exposure to the second and third trimesters O3 was significantly associated with lower birth weight and exposure to NO2 during the first trimester was linked to an increased risk of GDM. O3 exposures in the first trimester were connected to an elevated risk of gestational hypertension.
Benavides et al. (2022) [20]	Review article		USA	Identification of research questions commonly used for the evaluation of health impacts of urban policies at different stages of the policy process and discussion for future directions.	Evaluating the environmental health impacts of urban policies is critical for developing and implementing policies that lead to more healthy and equitable cities.
Hansel et al. (2022) [21]	Clinical trial		USA	116 randomized former smokers with moderate-severe COPD received active or sham portable particulate air cleaners and were followed for six months.	Environmental study among ex-smokers with COPD, especially for those who spend a lot of time at home, for the impact of air purifiers.
Lee et al. (2020) [22]	A systematic review, meta-analysis, and burden estimation study		UK	A systematic search of Ovid Embase, MEDLINE, and the Global Health and Web of Science for studies evaluating the association between exposure to household air pollution and adverse cardiorespiratory, pediatric health outcomes, and maternal health outcomes.	This analysis shows that household air pollution increases the risk of a wide range of adverse cardiorespiratory, pediatric, and maternal health outcomes.
Abubakar et al. (2022) [23]	Clinical trial		Malaysia	This study looked at heavy metal concentrations, concentrations in relation to threshold values, and assessments of risk for noncarcinogenic and cancer risk threats (p < 0.05).	The study concluded that informal e-waste burning has substantially helped in the relatively high levels of air pollution identified in the treatment points and in turn posed environmental and public health concerns to people around the area.
Ahmad et al. (2021) [24]	Clinical trial		Pakistan	The present study aimed to determine the composition of toxic metals (Cr, Mn, Cu, As) and heavy metals (Cd, Ba, Hg, Pb) in soil and water by an inductively coupled plasma optical emission spectrometer (ICP-OES) (p < 0.05).	The present observations present useful information regarding the contamination of soil and water with toxic and heavy metals in an industrial area, which also has much significance in areas where there are inappropriate practices in solid waste management.
Marsigliante et al. (2023) [25]	Clinical trial		Italy	A sample of 139 boys and 171 girls, aged between eight and 10 years was selected from schools in three cities with a similar socioeconomic status. These public schools with attendance from 8:00 to 13:00 have not participated previously in health promotion programs (p < 0.05).	Integrating physical activity allows for a reduction in the BMI of children and increases the levels of physical well-being, cognitive functioning, academic performance, and mood improvement.
Ordóñez-Iriarte (2020) [26]	Review article		Spain	Psychiatric enviromics has been defined as the study of environmental conditions and processes that promote mental health or increase the risk of developing mental disorders.	Association between mental health and the environment.
Ramadan et al. (2021) [27]	Review article		Egypt	The direct effects of climate change mostly occur after acute weather events and include post-traumatic stress disorder, anxiety, substance abuse disorder, depression, and even suicidal ideation.	Climate-related catastrophes have major impacts on the mental well-being of the populations involved, causing surges in cases of depression, anxiety, and PTSD primarily.
Anabitarte et al. (2021) [28]	Clinical trial		Spain	The participants were 167 children of seven years old distributed in four schools with similar socio-economic status, and ADHD symptomatology not diagnosed (p < 0.05).	This research is testing empirically whether exposure to a green environment improves attention in school children.
Mandolesi et al. (2018) [29]	Systematic review		Italy	Much evidence shows that physical exercise (PE) is a strong gene modulator that induces structural and functional changes in the brain, determining enormous benefits on both cognitive functioning and well-being.	Effects of physical exercise on cognitive functioning and well-being. Biological and psychological benefits. A growing body of literature indicates that both chronic and aerobic PE can achieve similar benefits.

Discussion

Although in recent years there have been a number of studies dealing with the effects of the environment on the health of vulnerable groups, it appears from the present study that the importance of this connection, as well as the necessity of appropriate information, education, and prevention in public health, has not been emphasized and assimilated into social perception.

This paper summarizes the results of different types of studies, from different countries, with different cultural backgrounds and large samples, on the serious negative effects of environmental degradation on the physical, psychological, and social health of children and their quality of life and the increase in morbidity and mortality due to these effects (Table 1).

This study also notes the gap in current literature and research, for information, recognition, and prevention in health services, but also for the lack of health policies and education in environmental and public health.

The devastating health effects of long-term exposure of children, even prenatally, to air pollution, are documented, and the connection is shown between PM2.5, PM10, O3, and NO2 with all respiratory infections, the risk of childhood leukemia, childhood asthma, and allergic diseases.

Furthermore, the relationship between improper solid waste management and public health problems is highlighted, including ground and water contamination, as well as the connection between indoor pollution in homes with a multitude of chronic respiratory and skin diseases.

The connection between mental health and the environment is emphasized, especially in children, as physical activity and physical exercise in a clean outdoor environment appear to result in biological and psychological benefits, reduce body mass index, contribute to physical and mental well-being, promote better psychological mood, and improve children's cognitive function and academic performance.

The right to health, especially of future generations, in today's era of the climate crisis, is an obligation of states, and information through educational programs in schools, mass media, and social media, on the connection between environmental degradation and public health and the increase in morbidity and mortality, especially of vulnerable groups, from a multitude of chronic diseases, is immediately necessary and required by current developments.

According to the United Nations Sustainable Development Goals, by 2030, it is necessary to ensure a substantial reduction in the number of deaths and illnesses caused by hazardous chemicals, pollution, and contamination of air, water, and land, as well as reducing premature mortality from non-communicable diseases by one-third through prevention and treatment and promoting mental health, well-being, and quality of life. As new scientific evidence is constantly generated, the air quality guidelines published by the WHO should be periodically reviewed and updated, following a multidisciplinary collaboration between health professionals and environmentalists to design new environmental policies.

## Conclusions

Policies and investments supporting cleaner transport, energy-efficient homes, power generation, better industry, and municipal waste management would reduce key sources of outdoor air pollution. Access to clean household energy would also greatly reduce ambient air pollution in some regions. There is a constant need to highlight the connection between the environment and public health as well as the health impacts on vulnerable populations from incorrect or inadequate approaches to managing environmental issues.

In conclusion, it needs to be widely known through all means of communication that children in particular can have life-long effects on their health and educational prospects from environmental degradation and therefore there is a need for monitoring, assessment, and special planning for their well-being.
